# Supplementary figures and images for: An immunological autobiography: my year as a COVID-19 vaccine trial participant
Source: NPJ Vaccines. 2022 Jul 18;7:80. doi: 10.1038/s41541-022-00502-z (PMC9293989; doi:10.1038/s41541-022-00502-z)

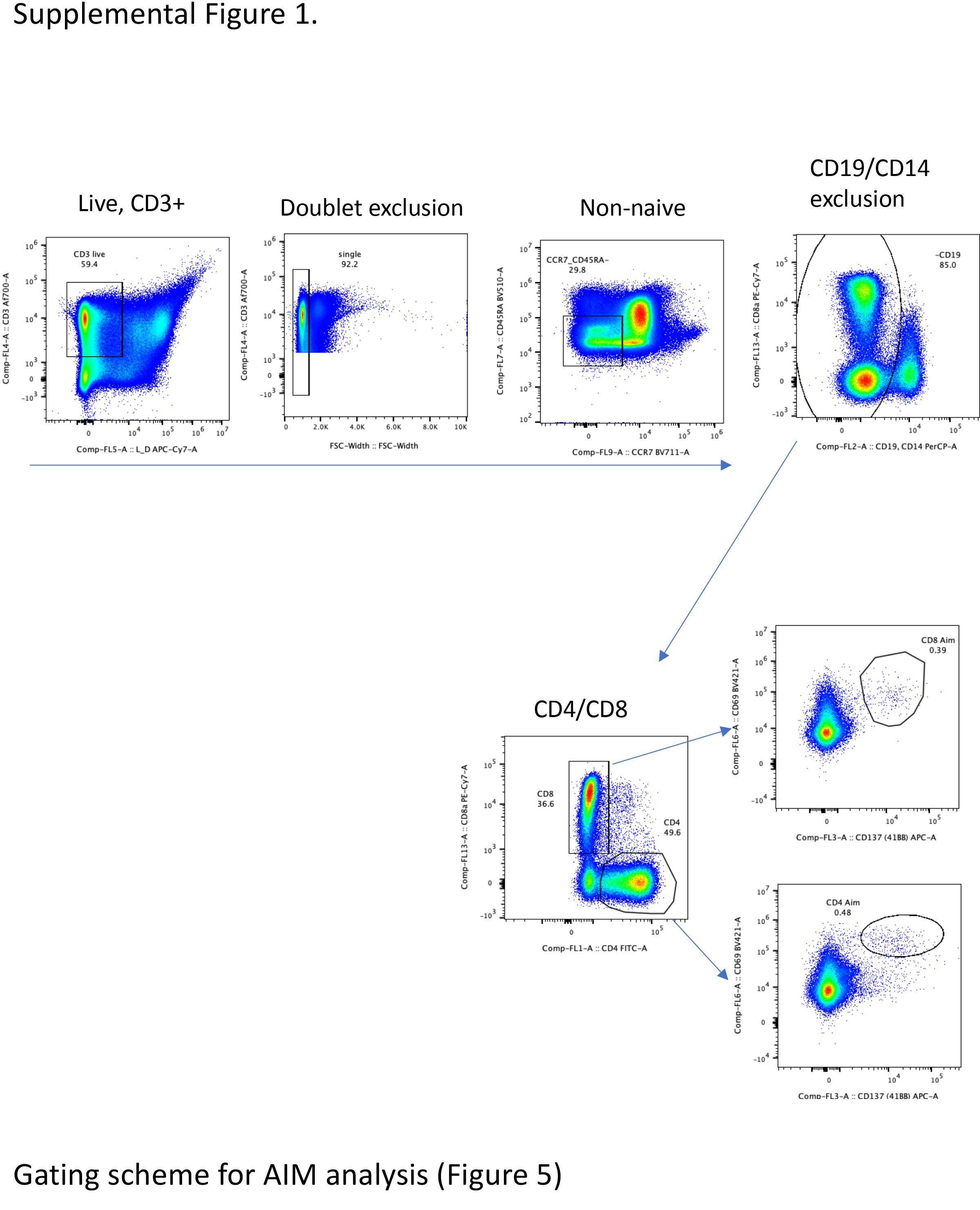

Supplement: Supplementary file 1 — Supplementary Figure 1 [file 41541_2022_502_MOESM1_ESM.jpg]
